# The relationship between personality and short video addiction among college students is mediated by depression and anxiety

**DOI:** 10.3389/fpsyg.2024.1465109

**Published:** 2024-10-28

**Authors:** Lei Zhang, Xing-feng Zhuo, Kai Xing, Yu Liu, Fang Lu, Jia-yi Zhang, Zheng-dong Qi, Li Zhang, Zheng-hong Yu, Chun-rong Gu

**Affiliations:** ^1^Department of Rheumatology and Immunology, Jinling Hospital, Affiliated Hospital of Medical School, Nanjing University, Nanjing, China; ^2^School of Early-Childhood Education, NanJing XiaoZhuang University, Nanjing, China; ^3^Jinling Hospital, Affiliated Hospital of Medical School, Nanjing University, Nanjing, China; ^4^Software Institute, Nanjing University, Nanjing, China; ^5^School of Information Engineering, Nanjing XiaoZhuang University, Nanjing, China; ^6^School of Languages, Literacies and Translation, Universiti Sains Malaysia, Penang, Malaysia; ^7^Binhai County People's Hospital, Yancheng, China

**Keywords:** college students, personality, short video addiction, depression, anxiety

## Abstract

**Background:**

Short video addiction (SVA) among college students is influenced by personality traits, namely, neuroticism and agreeableness. However, the role of depression and anxiety as mediators remains unclear.

**Objective:**

This study aims to explore the mediating role of comorbid depression and anxiety in the relationship between different dimensions of university students’ personalities and SVA.

**Methods:**

The SPSS PROCESS was utilized to analyze data from 804 university students across seven universities in China.

**Results:**

The findings show that neuroticism, agreeableness, and extraversion in the personalities of Chinese university students are all significantly linked to SVA; neuroticism and agreeableness in the personalities of university students have a greater impact on SVA; both neuroticism and agreeableness can first induce depression and then lead to anxiety and SVA, whereas only agreeableness can first lead to anxiety and then result in depression and SVA.

**Conclusion:**

This study uncovers the intricate relationship between personality traits and SVA among college students, emphasizing depression and anxiety as critical chain mediators in this relationship. It reveals that neuroticism and agreeableness significantly influence SVA through specific pathways involving depression and anxiety, indicating that interventions targeting these traits are essential.

## Introduction

Short video addiction (SVA) is a concept derived from internet addiction and addiction to mobile social apps. This study defines SVA as a chronic or periodic state of fascination caused by the repetitive use of short video apps, which generates a strong, persistent sense of need and dependence, psychologically and behaviorally (Qin et al., 2019; Li et al., 2021).

In recent years, the addiction of college students to short videos has become increasingly pronounced. As early as 2015, in a Pew Research poll, nearly half of Americans stated they “could not live without a smartphone” (Elhai et al., 2016)”. Many people frequently experience the illusion of phone vibrations even without receiving calls, revealing a significant impact of over-reliance on phones in daily life, with SVA being a typical manifestation. For instance, approximately 22% of TikTok users use the app for over an hour daily (Zhang et al., 2019). Such excessive use can lead to numerous psychological issues, such as being easily distracted, poor time management, and reduced study time—typical characteristics of addiction.

College students, as the primary users of internet applications, are particularly at risk of developing mobile app addiction, including SVA. Despite the rise in internet usage among teenagers, their needs and concerns regarding digital apps and platforms have been largely overlooked. In fact, nearly half of teenagers visit various social websites daily, including spending an average of more than one hour per day viewing short videos. Some studies found that up to 80.97% of Chinese college students view short videos (Xie and Jia, 2021; Zeighami et al., 2021). Clearly, this reliance on mobile phones has impacted the physical, psychological, and cognitive well-being of young people, including college students.

Given these observations, researchers have increasingly focused on the detrimental effects of excessive smartphone use on college students’ mental health and academic performance. Therefore, a comprehensive study of college students’ SVA, especially its causes, effects, and corresponding intervention strategies, is essential for building a healthier digital environment and ensuring the well-being of young people.

### Short video addiction and personality

Personality traits have been shown to influence susceptibility to SVA. A study by Zeighami et al. (2021) found that personality traits, namely neuroticism and openness to experience had a significant effect on addiction susceptibility. Extensive research has reported a significant correlation between behavioral addictions and elevated levels of personality traits in both treatment and community-based studies (Passanisi et al., 2022; Bağcı and Horzum, 2022; Dudfield et al., 2023). For instance, addiction to social media has been linked to individual characteristics of users, such as higher levels of neuroticism and lower levels of extraversion, which have been identified as predictors of social media addiction (Blackwell et al., 2017).

Personality plays a key role in how individuals mange stress and their tendencies toward addictive behaviors. For instance, shyness may be a significant predictor of internet and mobile phone addiction (Han et al., 2017; Tian et al., 2017). Individuals with a more shy personality are more likely to resort to addictive behaviors when using the internet (such as short video applications) to cope with stress. Additionally, a typical manifestation of shyness is a lack of social skills, which may also increase the chances of internet addiction (Iranmanesh et al., 2021). Mobile short video applications, with their relaxed content format, may offer users the possibility of stress relief anytime and anywhere. Receiving short-term rewards is becoming increasingly prevalent among users (Brand et al., 2016) and may exacerbate the SVA among teenagers (Dubey et al., 2020). Typically, individuals use short video applications as a temporary escape from the negative impacts of stress in the real world (Chu et al., 2020).

Mobile phone addiction has also been consistently linked to neuroticism (Giota and Kleftaras, 2013; Marciano et al., 2021; Montag and Markett, 2024). Individuals with higher levels of loneliness often turn to social media to compensate for the intimacy and sense of belonging they lack in real-world interactions (Ho et al., 2017). High levels of neuroticism are associated with an increased likelihood of social isolation (Whaite et al., 2018). Those with high neuroticism may develop anxiety about their social relationships, leading to a fear of isolation (Smith et al., 2017). To counteract these fears, individuals with high neuroticism may increase their media use, seeking virtual interactions that provide a sense of community and belonging (Xu et al., 2016; Blackwell et al., 2017).

Adolescents are in a critical period of personality development and are susceptible to becoming a high-risk group for SVA (Deng et al., 2017; Sha and Dong, 2021; Liu et al., 2021). They rely on establishing new social connections to maintain mental health, and the inability to create such connections may lead to low self-esteem, loneliness, and psychological distress (Tanta et al., 2014). Deep immersion in the flow experience of short videos may also indirectly influence students’ learning motivation and well-being through addictive behaviors (Ye et al., 2022), ultimately exerting a negative impact on their academic performance (Ye et al., 2023a). Moreover, many short video apps utilize personalized algorithm recommendations. Such personalized recommendation mechanisms allow these apps to continuously provide users with content tailored to their preferences, increasing their dependence on short videos and exacerbating the likelihood of addiction (Zhang et al., 2019). The personalized algorithm may also explain the higher failure rate faced by teenagers when trying to recover from SVA (Humphreys et al., 2017). Therefore, research and intervention regarding teenagers’ SVA are crucial to prevent further adverse impacts on their physical and mental health (Ye et al., 2023b; Ye et al., 2024). Once engrossed in short videos, teenagers might face functional brain changes and poorer mental health, intensifying their dependence on short videos and forming a vicious cycle (Mohammed et al., 2020).

### Depression and anxiety act as mediators

Depression and anxiety are closely linked to personality. Depression, also known as clinical depression or major depressive disorder, is a common mental health condition. It is characterized by persistent and long-lasting feelings of sadness. People with depression often lose interest or pleasure in activities they once enjoyed. It can also cause a range of physical and cognitive symptoms. Anxiety is an emotional experience, often characterized by excessive worry, tension, and unease about potential threats, dangers, or future events (American Psychiatric Association, 2013). Individuals with elevated levels of neuroticism are more susceptible to experiencing these emotional disturbances, thereby increasing the likelihood of engaging in addictive behaviors (Lahey, 2009). Conversely, agreeableness is positively associated with robust social support systems. Research has shown that individuals with higher levels of agreeableness are more adept at managing stress, thereby reducing the likelihood of experiencing depression and anxiety (Jensen-Campbell and Graziano, 2001; Soto and John, 2017). This has been supported by subsequent research. A meta-analysis by Lyon et al. (2021) of 408 related studies also found that agreeableness was significantly negatively correlated with anxiety. Additionally, a study involving 323 Chinese college students also found that neuroticism, conscientiousness, and agreeableness significantly predicted anxiety in this population (Liu et al., 2023).

Previous research has highlighted the correlation between neuroticism and both depression and anxiety (Sailo and Lalremruati, 2020). A meta-analysis of 175 studies on personality traits and common affective disorders found that neuroticism was positively correlated with anxiety while openness and agreeableness were unrelated to almost all affective disorders (Kotov et al., 2010). This has been supported by subsequent research. Later studies confirmed that neuroticism increased social isolation and worsened anxiety (Whaite et al., 2018). Moreover, Gong et al. (2020) found that neuroticism was positively associated with depressive symptoms.

Depression and anxiety have potential links to SVA. Flores Mosri (2019) suggests that depression forms the foundation for addictive disorders. Several studies that show a positive correlation between the use of social networking sites and depression support this notion (Davila et al., 2012; Chu et al., 2020). Studies have shown that short video overuse can exacerbate depressive symptoms, and depressive symptoms can also prompt individuals to use short video platforms more frequently (Zhang et al., 2024). This two-way relationship suggests that depression plays a role in the development of SVA.

A year-long longitudinal study by Kraut et al. (1998) observed that increased feelings of depression and loneliness were more common among those who spent more time online. Thus, depression and social anxiety have been reported repeatedly for excessive internet use (Ho et al., 2014). This suggests that negative emotions could lead to a heightened dependence on the internet, mobile phones, and short videos (Taipale, 2017). Supporting this, research by Elhai et al. (2016) and Flores Mosri (2019) has demonstrated a significant relationship between internet addiction and both depression and anxiety. It appears, therefore, that a maladaptive cycle is at play, where depression and anxiety not only result from but also contribute to further internet addiction, as noted by Mohammed et al., (2020).

Similarly, anxiety has a significant connection to addiction. For example, Cheever et al. (2014) and Clayton et al. (2015) found that many participants experienced increased heart rate and blood pressure when separated from their smartphones. During addiction withdrawal, individuals tend to undergo an unpleasant subjective experience that reinforces drug use. The association between smartphone use social anxiety may increase the risk of SVA among adolescents (Dey et al., 2019).

### Current study

**Figure 1:**
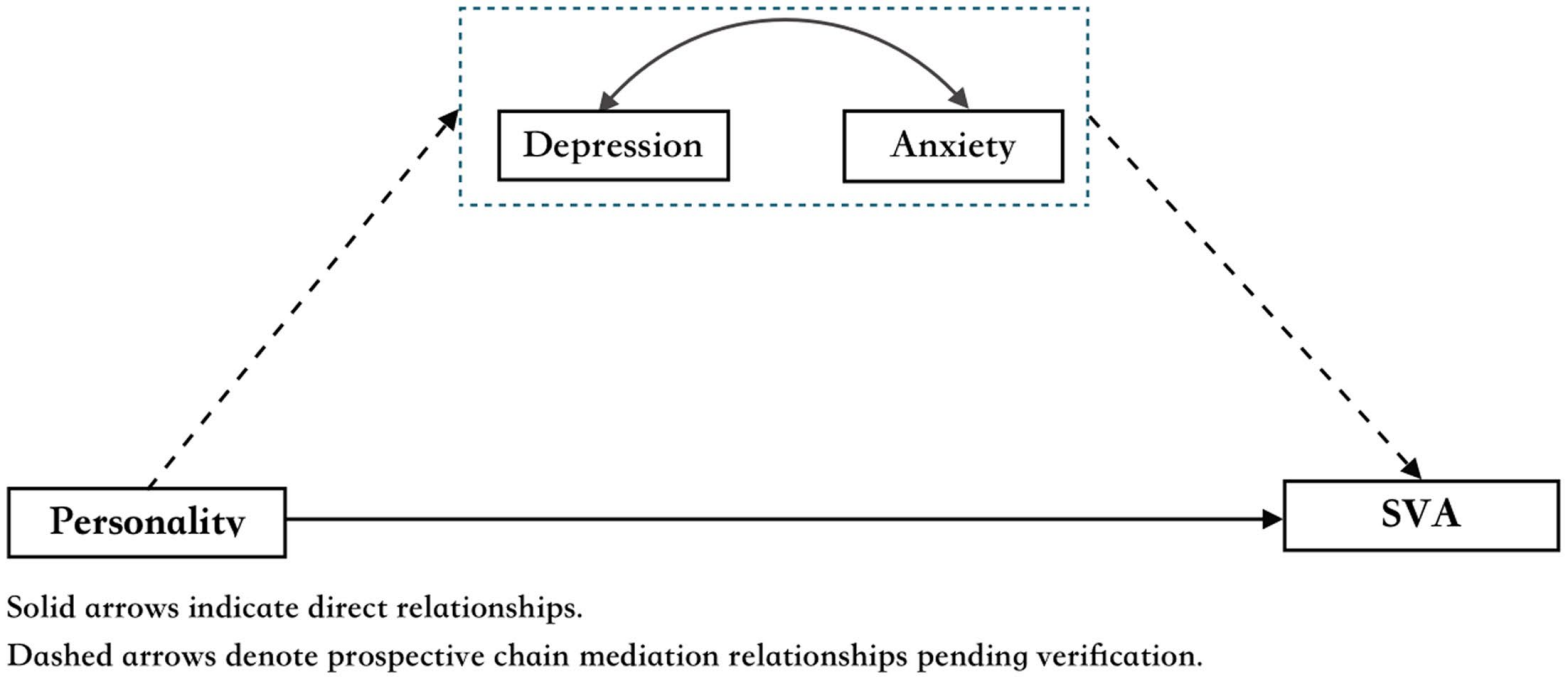
The framework of the research study.

Despite research on the relationship between personality traits and SVA, limited studies have explored the mediating roles of depression and anxiety in this dynamic (Gongyu et al., 2024). This study aims to bridge this gap by investigating how these emotional states mediate the effects of personality on SVA among college students. Because of the strong comorbidity between depression and anxiety (Kessler and Wang, 2008; McTeague et al., 2017; Hettema, 2008; Goodkind et al., 2015), depressive disorders may induce anxiety, and the reverse is also true. Therefore, we hypothesized that neuroticism promotes SVA by increasing depression and anxiety, whereas agreeableness reduces the risk of addiction by alleviating these negative emotions. We separately discuss the different roles of depression and anxiety in the connection between personality and SVA in college students, with the aim of elucidating the link between personality and SVA (see Figure 1). We hypothesize that:

1. There is a significant correlation between neuroticism, agreeableness, and SVA among college students;
2. Depression and anxiety act as chained mediators in the relationship between neuroticism, agreeableness, and SVA in college students.

Building on the theoretical framework that links personality traits, depression, and anxiety to SVA, it becomes crucial to empirically explore these relationships in a real-world context. To this end, our study employed a methodologically rigorous approach to examine the interaction of these variables among Chinese college students.

## Methods

### Participants and procedures

The current study used random sampling on campus to recruit 804 participants from seven universities across China between January 2023 and June 2023. Among these universities, two were dedicated to teacher education, three specialized in science and engineering, and two were comprehensive institutions. Of the recruited participants, 35.7% (287) were male, and 64.3% (517) were female. This imbalance in gender distribution may lead to response bias. As a result, our findings might not fully represent the broader population of university students. To address this issue, we included gender as a control variable in our analysis to account for its potential impact on the results. All participants were undergraduate students aged between 18 and 22 years. Prior to data collection, the study was approved by the Ethics Committee of Psychological Research of the authors’ institution. We emphasized the anonymity of the study and obtained consent from all participants. Participants completed the questionnaires in a quiet classroom or online and were free to withdraw from the study at any time.

### Materials

Big Five personality traits were assessed using the Chinese Big Five Personality Inventory Brief Version (Wang et al., 2011), a 40-item self-report questionnaire measuring aspects of personality including neuroticism, openness, conscientiousness, extraversion, and agreeableness. Participants rated each item on a scale from 1 (did not apply to at all) to 6 (applied to me very much). Cronbach’s alpha reliability coefficient was 0.83 for neuroticism, 0.81 for conscientiousness, 0.76 for agreeableness, 0.85 for openness, and 0.73 for extraversion.

Depression was assessed using the Chinese version of the Self-Rating Depression Scale (SDS) developed by Zung (1965). This scale includes 20 items, with a four-point rating scale (ranging from “a little of the time” to “most of the time”). A higher score indicates a higher level of depression. The Cronbach’s alpha reliability coefficient for the current sample was 0.829.

Anxiety was assessed using the Chinese version of the Self-Rating Anxiety Scale (SAS) developed by Zung (1971). This scale includes 20 items, with a four-point rating system (ranging from “a little of the time” to “most of the time”). A higher score indicates a higher level of anxiety. The Cronbach’s alpha reliability coefficient for the current sample was 0.874.

The College Students’ Short Video Addiction Questionnaire (Qin et al., 2019) was used to assess the level of SVA among college students. This scale includes 14 items, and is divided into four dimensions: lack of control, withdrawal, escapism, and inefficiency. Participants rated each item using a 5-point rating system, ranging from 1 (strongly disagree) to 5 (strongly agree). A higher score indicates a stronger possibility of SVA. In this study, the internal consistency coefficients for the dimensions of the SVA scale were 0.475 for lack of control, 0.844 for withdrawal, 0.864 for escapism, and 0.815 for inefficiency. The overall internal consistency reliability coefficient was 0.887. The KMO value for the scale was 0.896, and Bartlett’s test of sphericity was significant, indicating good structural validity for the questionnaire.

### Statistical analysis

SPSS 27 was used for descriptive analyses, correlation analyses, and regression analyses. In this mediation relationship study, we used specific dimensions of personality as independent variables, depression and anxiety as mediating variables, and SVA as the dependent variable. PROCESS program developed by Hayes (2018) has a total of 74 models, among which Model 6 best fits our research hypothesis as illustrated in Figure 1. The significance level of the mediating effect was tested using the bias-corrected non-parametric percentile Bootstrap method (*n* = 5,000).

## Results

### Descriptive statistics and correlation analysis

As shown in Table 1, there was a significant correlation between SVA and depression, anxiety, gender, neuroticism, agreeableness, and extraversion. Depression was significantly related to anxiety, neuroticism, conscientiousness, agreeableness, openness, and extraversion, while anxiety was significantly associated with neuroticism, conscientiousness, and agreeableness. This result supports Hypothesis 1.

**Table 1:** Correlation matrix of all variables.

Variables	1	2	3	4	5	6	7	8	9
1. SVA	1								
2. Depression	0.299**	1							
3. Anxiety	0.328*	0.730**	1						
4. Neuroticism	0.368**	0.538**	0.580**	1					
5. Conscientiousness	0.033	−0.259**	−0.081*	0.076*	1				
6. Agreeableness	0.074*	−0.214**	−0.106**	0.048	0.397**	1			
7. Openness	0.043	−0.229**	0.000	0.061	0.499**	0.381**	1		
8. Extraversion	0.137**	−0.169**	−0.011	−0.070*	0.288**	0.284**	0.516**	1	
9. Gender	0.092**	−0.016	−0.019	0.068	0.137*	0.155**	0.046	0.068	1

*N* = 804. *p < 0.05, **p < 0.01.

As shown in Table 2, regression analyses revealed that neuroticism (*β* = 0.569, *p* < 0.001), agreeableness (*β* = 0.141, *p* < 0.05), and extraversion (*β* = 0.238, *p* < 0.001) can predict SVA. Among them, neuroticism (13.6%) and agreeableness (6%) have a greater impact on SVA. Therefore, we will discuss the mediating relationships of depression and anxiety between neuroticism, agreeableness and SVA.

**Table 2:** Linear regression of the Big Five personality dimensions.

Predictor variables	SVA		R^2^	Durbin-Watson
	*β*	*t*		
Neuroticism	0.569	11.217***	0.136	1.997
Conscientiousness	0.058	0.922	0.01	1.998
Agreeableness	0.141	2.115*	0.06	1.998
Openness	0.070	1.206	0.002	2.005
Extraversion	0.238	3.912***	0.019	2.003

*p < 0.05, ***p < 0.001.

### Mediation model analysis

To further explore the specific relationship between neuroticism, agreeableness, depression, anxiety, and addiction, we used the PROCESS macro to analyze the chain mediation effect. While controlling for gender, we analyzed the mediating role of depression and anxiety between neuroticism (or agreeableness) and SVA.

#### Pathway 1

Chain mediation of depression and anxiety for the neuroticism model.

**Figure 2:**
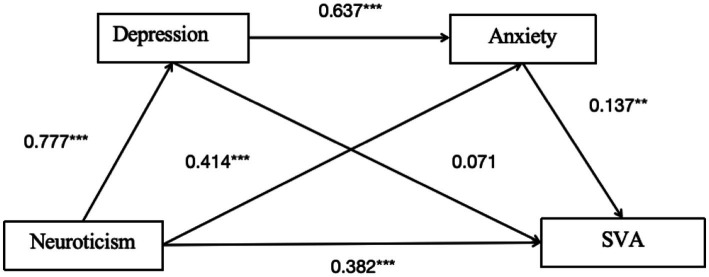
Mediation path diagram of depression and anxiety in pathway 1. **p < 0.01, ***p < 0.001.

As shown in Table 3 and Figure 2, the total effect of neuroticism on college students’ SVA is 0.562 [95% CI: 0.462, 0.661] indicating a significant total effect. The direct effect is 0.382 [95% CI: 0.258, 0.506], indicating a significant direct effect (Table 4).

**Table 3:** Regression analyses of the chain mediation model for pathway 1.

Predictor variables	Depression		Anxiety		SVA	
	*β*	*t*	*β*	*t*	*β*	*t*
Gender	−1.14	−1.781	−6.24	−1.206	1.759	2.415*
Neuroticism	0.777	18.186***	0.414	9.776***	0.382	6.066***
Depression			0.637	21.627***	0.071	1.362
Anxiety					0.137	2.752**
R^2^	0.292		0.583		0.163	
*F*	165.512		373.167		38.976	

*p < 0.05, **p < 0.01, ***p < 0.001.

**Table 4:** Indirect effects of agreeableness on SVA for pathway 1.

Mediation path	Effect	BootSE	Boot CI (95%)	Relative effect
Total effect	0.562	0.051	[0.462, 0.661]	
Direct effect	0.382	0.063	[0.258, 0.506]	
Total indirect effect	0.180	0.044	[0.095, 0.268]	100%
1. Neuroticism→Depression→SVA	0.055	0.045	[−0.029, 0.151]	31%
2. Neuroticism→Anxiety→SVA	0.057	0.025	[0.009, 0.108]	31%
3. Neuroticism→Depression→Anxiety→SVA	0.068	0.027	[0.012, 0.120]	38%

For the Indirect Effect 1 (neuroticism-depression-SVA), the indirect effect is 0.055 [95% CI: −0.029, 0.151], suggesting the mediation effect is not significant.

For the Indirect Effect 2 (neuroticism-anxiety-SVA), the indirect effect is 0.057 [95% CI: 0.009, 0.108], indicating a significant mediation effect.

For the Indirect Effect 3 (neuroticism-depression-anxiety-SVA), the indirect effect is 0.068 [95% CI: 0.012, 0.120], suggesting a significant chain mediation effect of depression and anxiety on the relationship between personality characteristics and SVA.

#### Pathway 2

Chain mediation of depression and anxiety for the agreeableness model.

**Figure 3:**
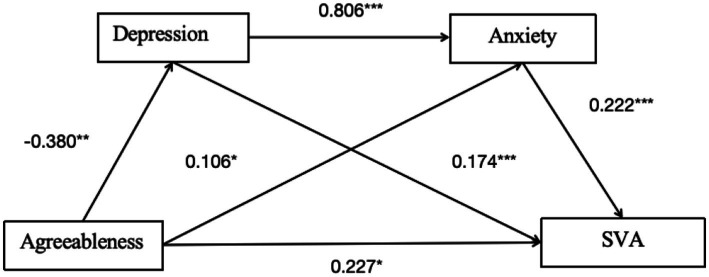
Mediation path diagram of depression and anxiety in pathway 2. *p < 0.05, **p < 0.01, ***p < 0.001.

As shown in Table 5 and Figure 3, the overall effect of agreeableness on SVA was 0.117 [95% CI: −0.015, 0.249], which is not significant. However, the direct effect is 0.227 [95% CI: 0.101, 0.354], indicating a significant direct effect (Table 6).

**Table 5:** Regression analyses of the chained mediation model for pathway 2.

Predictor variables	Depression		Anxiety		SVA	
	*β*	*t*	*β*	*t*	*β*	*t*
Gender	0.360	0.496	−0.354	−0.643	1.788	2.401*
Agreeableness	−0.380	−6.192***	0.106	2.222*	0.227	3.524*
Depression			0.806	30.100***	0.174	3.300***
Anxiety					0.222	4.643***
*R*^2^	0.046		0.536		0.138	
*F*	19.285		308.418		32.15	

*p < 0.05, ***p < 0.001.

**Table 6:** Indirect effects of agreeableness on SVA for pathway 2.

Mediation path	Effect	BootSE	Boot CI (95%)	Relative effect
Total effect	0.117	0.067	[−0.015, 0.249]	
Direct effect	0.227	0.065	[0.101, 0.354]	
Total indirect effect	−0.111	0.033	[−0.176, −0.048]	100%
1. Agreeableness→Depression→SVA	−0.066	0.025	[−0.122, −0.022]	60%
2. Agreeableness→Anxiety→SVA	0.023	0.017	[−0.005, 0.061]	−21%
3. Agreeableness→Depression→Anxiety→SVA	−0.068	0.022	[−0.115, −0.030]	61%

For Indirect Effect 1, agreeableness-depression-SVA, the indirect effect was −0.066 [95% CI: −0.122, −0.022], indicating a significant mediating effect.

For Indirect Effect 2, agreeableness-anxiety-SVA, the indirect effect was 0.023 [95% CI: −0.005, 0.061], indicating that the mediating effect is not significant.

For Indirect Effect 3, agreeableness-depression-anxiety-SVA, the indirect effect was −0.068 [95% CI: −0.115, −0.030], indicating a significant chain mediating effect of depression and anxiety on the relationship between agreeableness and SVA.

#### Pathway 3

Chain mediation of anxiety and depression for the neuroticism model.

**Figure 4:**
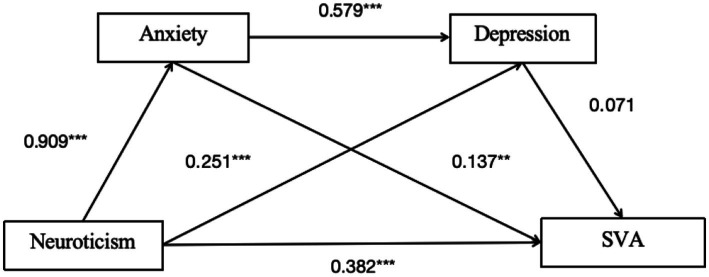
Mediation path diagram of anxiety and depression in pathway 3. **p < 0.01, ***p < 0.001.

As shown in Table 7 and Figure 4, the overall effect of neuroticism on SVA was 0.562 [95% CI: 0.462, 0.661], indicating that the overall effect is significant. The direct effect was 0.382 [95% CI: 0.258, 0.506], suggesting that the direct effect is significant (Table 8).

**Table 7:** Regression analyses of the chained mediation model for pathway 3.

Predictor variables	Anxiety		Depression		SVA	
	*β*	*t*	*β*	*t*	*β*	*t*
Gender	−1.328	−2.042*	−0.335	−0.679	1.759	2.415*
Neuroticism	0.909	20.283***	0.251	5.998***	0.382	6.066***
Anxiety			0.579	21.627***	0.137	2.752**
Depression					0.071	1.362
R^2^	0.340		0.553		0.163	
*F*	205.915		330.537		38.976	

*p < 0.05, **p < 0.01, ***p < 0.001.

**Table 8:** Indirect effects of neuroticism on SVA for pathway 3.

Mediation path	Effect	BootSE	Boot CI (95%)	Relative effect
Total effect	0.562	0.051	[0.462, 0.661]	
Direct effect	0.382	0.063	[0.258, 0.506]	
Total indirect effect	0.180	0.045	[0.091, 0.268]	100%
1. Neuroticism→Anxiety→SVA	0.124	0.050	[0.024, 0.222]	69%
2. Neuroticism→Depression→SVA	0.018	0.015	[−0.009, 0.051]	10%
3. Neuroticism→Anxiety→Depression→SVA	0.037	0.030	[−0.019, 0.098]	21%

For Indirect Effect 1, neuroticism-anxiety-SVA, the indirect effect was 0.124 [95% CI: 0.024, 0.222], indicating a significant mediating effect.

For Indirect Effect 2, neuroticism-depression-SVA, the indirect effect was 0.018 [95% CI: −0.009, 0.051], suggesting the mediating effect was not significant.

For Indirect Effect 3, neuroticism-anxiety-depression-SVA, the indirect effect was 0.037 [95% CI: −0.019, 0.098], indicating a significant chain mediating effect of anxiety and depression on the relationship between neuroticism and SVA.

Pathway 4: Chain mediation of anxiety and depression for the agreeableness model.

**Figure 5:**
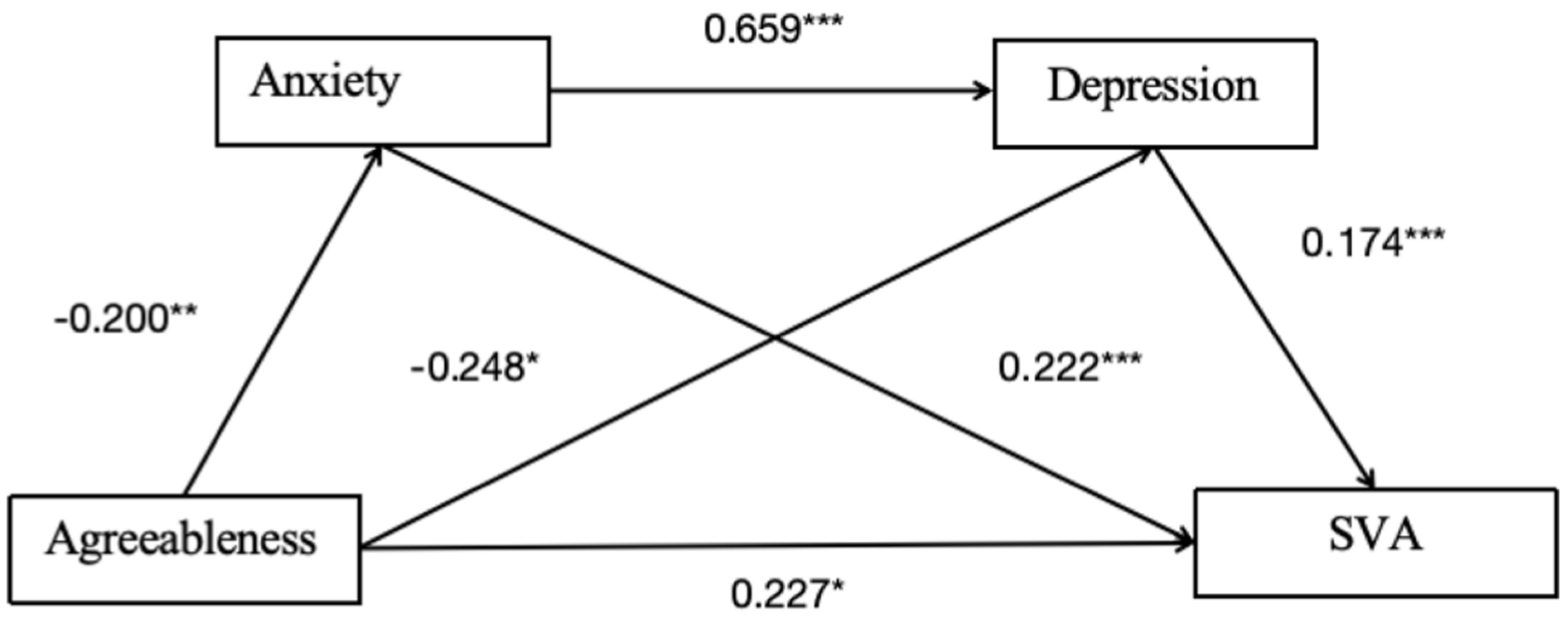
Mediation path diagram of anxiety and depression for pathway 4. *p < 0.05, **p < 0.01, ***p < 0.001.

As shown in Table 9 and Figure 5, the overall effect of agreeableness on college students’ SVA was 0.117 [95% CI: −0.015, 0.249], indicating that the overall effect is not significant. Additionally, the direct effect was 0.227 [95% CI: 0.101, 0.354], indicating that the direct effect was significant (Table 10).

**Table 9:** Regression analyses of the chained mediation model for pathway 4.

Predictor variables	Anxiety		Depression		SVA	
	*β*	*t*	*β*	*t*	*β*	*t*
Gender	−0.064	−0.079	0.402	0.808	1.788	2.401*
Agreeableness	−0.200	−2.955**	−0.248	−5.863***	0.227	3.524*
Anxiety			0.659	30.100***	0.222	4.643***
Depression					0.174	3.300***
R^2^	0.011		0.553		0.138	
*F*	4.513		329.392		32.15	

*p < 0.05, **p < 0.01, ***p < 0.001.

**Table 10:** Indirect effects of neuroticism on SVA for pathway 4.

Mediation path	Effect	Boot SE	Boot CI (95%)	Relative effect
Total effect	0.117	0.067	[−0.015, 0.249]	
Direct effect	0.227	0.065	[0.101, 0.354]	
Total indirect effect	−0.111	0.032	[−0.175, −0.050]	100%
1. Agreeableness→Anxiety→SVA	−0.044	0.021	[−0.092, −0.008]	40%
2. Agreeableness→Depression→SVA	−0.043	0.017	[−0.079, −0.014]	39%
3. Agreeableness→Anxiety→Depression→SVA	−0.023	0.013	[−0.053, −0.003]	21%

For Indirect Effect 1, agreeableness-anxiety-SVA, the indirect effect was −0.044 [95% CI: −0.092, −0.008], suggesting a significant mediating effect.

For Indirect Effect 2, agreeableness-depression-SVA, the indirect effect was −0.043 [95% CI: −0.079, −0.014], showing a significant mediating effect.

For Indirect Effect 3, agreeableness-anxiety-depression-SVA, the indirect effect was −0.023 [95% CI: −0.053, −0.003], indicating that the chained mediating effect of anxiety and depression on the relationship between agreeableness and SVA is significant.

Overall, these pathways reveal the complex interplay between neuroticism, agreeableness, depression, and anxiety in SVA. Our findings not only help clarify how neuroticism drives addictive behavior through depression and anxiety, but also illustrate how agreeableness reduces addiction risk by mitigating these negative emotions, allowing for a deeper exploration of how these results relate to existing addiction theories.

## Discussion

This study investigates the relationship between neuroticism, agreeableness, and SVA among college students, with a focus on the mediating role of depression and anxiety. The results reveal significant correlations between personality traits such as neuroticism, agreeableness, and extraversion, and SVA. These findings are consistent with the existing literature (Bianchi and Phillips, 2005; Augner and Hacker, 2012), which indicates that neuroticism and agreeableness significantly influence addictive behaviors, including digital media consumption. Importantly, our study discovered that depression and anxiety play a chain mediating role between personality traits and SVA. This suggests that individuals with higher levels of neuroticism may be more prone to experiencing elevated levels of depression and anxiety, which in turn increases their dependency on SVA, whereas higher agreeableness may reduce the impact of these negative emotions, thereby lowering the risk of addiction (Dal, 2018).

Results from the current study suggests that the influence of personality traits on SVA operates through three pathways: neuroticism-depression-anxiety-SVA, agreeableness-depression-anxiety-SVA and agreeableness-anxiety-depression-SVA. The results specifically elucidate the mechanisms by which neuroticism and agreeableness influence SVA. The findings of the study elucidate that the neuroticism-depression-anxiety-SVA and neuroticism-anxiety-SVA pathways demonstrate statistical significance, whereas the neuroticism-depression-SVA trajectory does not manifest as significant. This delineates anxiety as a pivotal mediator in the nexus between neuroticism and SVA. The validity of the neuroticism-depression-anxiety-SVA sequence can be attributed to the comorbid nature of depression and anxiety, suggesting that individuals with neurotic predispositions may be inclined to develop anxiety disorders following depressive episodes, subsequently precipitating SVA. That is, college students with high neuroticism may experience more depressive emotions, prompting them to feel anxious, which increases SVA. Individuals suffering from depression may engage in short video watching to temporarily relieve anxiety or discomfort (Elhai et al., 2019; Augner et al., 2023), as the instant happiness this provides fosters repeated desire, ultimately leading to addictive behavior (Nong et al., 2023). Additionally, highly neurotic people tend to be less confident in themselves (Ohana, 2016) and have a perfectionistic attitude (MacNicol et al., 2003). Thus, they feel more helpless, sad, and powerless when stressed. These emotions are linked to depression and anxiety, which further drives students who are already more susceptible to addictive behaviors (Sibilla et al., 2022), resulting in a cycle of increased mood symptoms and increased short video viewing. The personality structures examined in this study are defined as follows: Individuals with high levels of neuroticism often feel anxious, restless, sad, and have poor skills to cope with stress. Within the mediation analysis concerning the dimension of agreeableness, the pathways agreeableness-depression-anxiety-SVA and agreeableness-anxiety-depression-SVA were found to be statistically significant. This indicates that depression and anxiety both influence each other in their mediating roles between agreeableness and SVA, with similar magnitudes of effect observed in each pathway.

Conversely, at the same time, college students with high agreeableness may exhibit fewer anxiety or depressive emotions (Kaplan et al., 2015; Soto and John, 2017), which in turn reduces their likelihood of developing SVA. This may be because agreeable individuals are more optimistic, have more trust, empathy, and better psychological resilience (Marengo et al., 2022; Liu et al., 2023). Therefore, they experience fewer depressive and anxious emotions (Sailo and Lalremruati, 2020), are less reliant on short videos, and are less prone to SVA. The pathways of agreeableness-depression-anxiety-SVA and agreeableness-anxiety-depression-SVA in this study support this hypothesis, where agreeableness masks the mediating role of depression and anxiety.

From a theoretical standpoint, this study sheds light on the multi-pathway mechanisms through which personality traits influence addiction behaviors. This aligns with the Opponent Process Theory (OPT), which suggests that addictive behaviors are maintained through alternating cycles of positive and negative emotions (Tian et al., 2023). This perspective expands upon the emotional regulation theories within addiction psychology, suggesting that individuals with higher neuroticism may be more vulnerable to this emotional-addiction feedback loop due to their heightened sensitivity to negative affect (Liao, 2024). Understanding of the interplay between emotions and personality traits provides a more comprehensive view of the field of psychology than simple behavioral outcomes, and can help design better targeted intervention strategies.

## Conclusion

The findings of this study elucidate the mediating roles of depression and anxiety between neuroticism, agreeableness, and SVA, emphasizing the intricate interaction between mental health and digital media consumption. This suggests that interventions designed to mitigate SVA should also tackle potential mental health issues, tailored to the distinct dimensions of personality. In particular, stress management and coping strategies for students with high levels of neuroticism could be beneficial in reducing their risk of developing SVA. For individuals with high levels of neuroticism, effective interventions could include cognitive-behavioral therapy (CBT), mindfulness meditation, and emotional regulation training to manage stress and reduce dependency on short videos (Prayogi et al., 2024; Yan et al., 2024; Jiang and Yoo, 2024). Additionally, physical exercise has been shown to significantly alleviate symptoms of depression and anxiety, which in turn could help decrease SVA (Jianfeng et al., 2024). These strategies should be personalized to align with the individual’s specific personality traits to maximize their effectiveness. Furthermore, this study touches on the role of digital media in modern life, especially its impact on college students, thus sparking important discussions on balancing the beneficial and potentially harmful use of digital platforms.

Although this study provides an in-depth exploration of the factors influencing SVA, several limitations should be acknowledged. First, the population used in this study exhibited a gender imbalance, which may introduce response bias and limit the representativeness of the findings. Second, because of the cross-sectional design, we were unable to determine clear causal relationships. Finally, the specific cultural and demographic backgrounds of the participants may limit the generalizability of the findings. Future research should aim to recruit a more balanced and diverse population to improve representativeness and reduce potential biases in the findings. Additionally, future studies could utilize longitudinal designs to more accurately discern causal relationships explore these relationships across different cultural contexts or age groups, and consider other potential mediators or moderators to fully understand the complex factors affecting SVA.

In summary, this study offers important perspectives on understanding how personality traits, depression, and anxiety interact and influence SVA among college students. These findings not only help deepen our understanding of the psychological basis of SVA, but they can also be used to guide the development of effective prevention and intervention strategies.

## Data Availability

The raw data supporting the conclusions of this article will be made available by the authors, without undue reservation.
